# Nordic hamstring exercises in functional knee rehabilitation after anterior cruciate ligament reconstruction: a prospective, randomised, controlled study

**DOI:** 10.1038/s41598-023-45817-6

**Published:** 2023-11-03

**Authors:** JiaWei Chen, TianYu Wu, Ying Guo

**Affiliations:** 1 Hunan Mechanical Electrical Polytechnic, Changsha, 410000 Hunan China; 2 Beijing Sport University, Beijing, 100010 China; 3 The People’s Liberation Army Joint Logistic Support Force Sanya Rehabilitation and Recuperation Center, Sanya, 572000 Hainan China; 4 Qiongzhong People’s Hospital of Li and Miao Autonomous Country, Qiongzhong County, 572923 Hainan China

**Keywords:** Anatomy, Musculoskeletal system, Ligaments

## Abstract

To study the effect of using Nordic hamstring exercise method on muscle strength and knee joint stability of patients after ACL reconstruction. 60 patients admitted to our hospital for ACL reconstruction were randomly divided into a test group (n = 30, applying Nordic hamstring exercise) and a control group (n = 30, applying conventional rehabilitation training methods), and the difference in the circumference of the thighs of the patients in the two groups was analysed after training, and the peak torque (PT), total torque (PT), and flexion and extension strength were measured by using the Biodex system3 Multi-joint Isokinetic Testing System at knee joints with an angular velocity of 60°/s and 120°/s. The peak torque (PT), total work (TW), and average peak torque (AVG PT) were measured by extension and flexion strength at angular velocity, and the Lysholm Knee Score was used to assess the knee function of 60 patients. There was no difference in the difference in thigh circumference between the two groups before surgery (P > 0.05); the difference in thigh circumference between the patients in the test group at 12 and 24 weeks after surgery was (− 0.35 ± 0.22) cm and (0.12 ± 0.03) cm, respectively, which were higher than those in the control group, (− 0.51 ± 0.15) cm and (− 0.41 ± 0.34) cm (P < 0.05). At the 12th and 24th postoperative weeks, the popliteal muscle strength of both groups was improved compared with that before surgery; among them, by comparing the popliteal peak moments with different angular velocities, the ratio of popliteal peak moment on the affected side/peak moment on the healthy side of the popliteal muscle of the experimental group was significantly higher than that of the control group, and the difference was significant (P < 0.05), but there was still a gap compared with that of the healthy side. The Lachmen test and the anterior drawer test were negative in the test and control groups at the 24th postoperative week of review, and the anterior tibial shift was < 5 mm in the KT-1000 test, and the difference in the anterior shift was < 3 mm compared with the healthy side, and there was no significant difference between the two groups. By Nordic hamstring exercise can make patients after knee ACL reconstruction reduce patient pain, accelerate the recovery of knee function, improve the swelling of the lower limb, reach the level of flexor strength of the healthy side within 24 weeks, and can increase the stability of the knee joint.

## Introduction

Anterior cruciate ligament (ACL) injuries are among the more serious knee sports injuries and have become a rehabilitation problem in sports medicine, orthopaedics and rehabilitation medicine^1^. The ACL is the primary restrictive ligament that prevents the tibia from sliding forward and internally rotating relative to the femur^2^. Fracture of the ACL allows forward displacement and internal rotation of the tibia, which can cause recurrent episodes of knee instability and damage to the meniscus and knee surface^3^. Arthroscopic reconstruction of the ACL and early postoperative intervention in rehabilitation are now commonly used methods to consolidate surgical results and improve knee function after ACL rupture. Complications such as knee pain, combined dysfunction, oedema and muscle atrophy often occur in the post-operative period, so a good post-operative training system is needed to help patients recover^4,5^. Studies have shown that post-operative rehabilitation greatly influences the final outcome of surgery and that muscle strength rehabilitation is an important component of post-operative rehabilitation for ACL reconstruction^6^.

With the development of urbanisation, many scholars^7^, both at home and abroad, use different methods of postoperative rehabilitation training. Baroni et al.^8^conducted a literature review on the methods used for centrifugal training of knee extensors in healthy subjects and the assessment of adaptations in strength, activation and structure of the muscle groups responsible for knee extension. Seventy-five studies were meticulously analysed and 30 of them were reviewed. The results show that the increase in strength after centrifugal training of knee extensors is caused by structural adaptation of the nervous system. It may help athletes in the prevention of muscle injuries and rehabilitation after muscle injuries. Wen Jie et al.^9^showed that isometric centrifugal training of hamstrings could improve the active knee extension angle, muscle strength of flexion-related muscle groups and knee function in ACL reconstruction patients. In addition to the ACL, the hamstrings also play an important role in preventing anterior tibial translation. Towards the end of a fast walking stride, the hamstrings produce an effective centrifugal contraction to decelerate the calf swing^10^, thus preventing excessive anterior tibial momentum from adversely affecting the ACL or the graft. However, postoperative hamstring muscle strength training has not been given enough attention, and most of the training methods focus on centripetal contraction^11^, which may cause an imbalance in knee flexion and extension muscle strength and result in ACL grafts not being able to maintain in the appropriate tension range during high-intensity exercise, resulting in secondary injuries^12^. Centrifugal training, on the other hand, is effective in improving joint mobility^13^, muscle strength and neuromuscular control^14^, thereby reducing injury risk and improving joint function.

Nordic hamstring exercise (NHE) is a knee-led, self-weighted, centrifugal exercise proposed by Mjolsnes et al.^15^in 2004, and has been included in the "FIFA11+" comprehensive "FIFA11+" programme by the FIFA Medical Research Centre (F-MARC). Warm-up exercises. Studies have shown that 6–10 weeks of Nordic hamstring exercises can effectively improve hamstring centrifugal muscle strength and significantly reduce injury and recurrence rates^16–18^.Nordic Hamstring Exercise focuses on centrifugal training for the purpose of activating the muscle, reducing the risk of endurance injuries regarding the load tissue^19^, and providing strengthening of the knee joint stability, preventing sports injuries and improving lower limb mobility^20^. In view of this, the present study focused on the effects of Nordic hamstring exercises on patients after ACL reconstruction of the knee, by evaluating the difference in thigh circumference between the two groups of patients after training, and measuring their peak torque (PT) by using the Biodex system3 Multi-Joint Isokinetic Testing System to measure the extension and flexion strengths of the knee joints at angular velocities of 60°/s, 120°/s, Total Work (TW), Average Peak Moment (AVG PT) and other indexes, in order to find a more effective way for patients after ACL reconstruction of the knee. The results are summarised as follows.

## Materials and methods

### General information

Sixty patients were selected after arthroscopic autologous hamstring tendon reconstruction at ACL injury from August 2018 to January 2020, and to avoid other factors interfering with the test results, the subjects selected were all patients with unilateral ACL rupture of the knee. Write informed consent was obtained from each participant before starting the study,which was approved by the Research Ethics Committee of Hunan Mechanical Electrical Polytechnic and in accordance with the Declaration of Helsinki. Sixty patients, all male, The randomised control table method was used to randomly divide into 2 groups of 30 cases each: (i) test group, age 29.3 ± 6.3 years, duration of disease 38.6 ± 13.5 day, 12 left knees and 18 right knees. (ii) Control group, age 31.7 ± 4.8 years, duration of disease 34.1 ± 11.7 day, 9 cases in the left knee and 21 cases in the right knee.

### Inclusion and exclusion criteria

#### Inclusion criteria

(1) Meet the diagnostic criteria for ACL injuries in the American Academy of Orthopaedic Surgeons' Guidelines for the Management of Anterior Cruciate Ligament Injuries^21^; (2) All were male, aged 18–58 years old, weighing 151–187 cm and 55–92 kg; (3) Unilateral anterior cruciate ligament rupture was detected and reconstructed using magnetic resonance imaging and other imaging means; (4) Positive Lachman or axial shift test; (5) No lower limb fracture; (6) No peripheral nerve injury; (7) No postoperative infection; (8) No cognitive dysfunction; (9) No respiratory and circulatory disorders; (10) Signed an informed consent form.

#### Exclusion criteria

(1) combined with posterior cruciate ligament rupture; (2) combined with tibial and fibular collateral ligament rupture; (3) combined with meniscus tear; (4) bone-patellar tendon-bone reconstruction of ACL and semitendinosus tendon reconstruction of ACL after surgery; (5) 1 month after the operation, the range of knee flexion and extension is still unable to reach 20°~80° or there is a suspicion of positive anterior drawer test; (6) unable to tolerate isokinetic centrifugal training of popliteus muscle; (7) patients with difficulty in reaching 120° of knee flexion angle due to obesity; (8) History of previous knee ligament injuries with a femoral footprint of < 14 mm; (9) comorbidities such as bone injuries, small intercondylar fossa and joint degeneration; and (10) osteoarthritis of the knee X-ray grading up to grade II, III, or IV lesions; (11) The person who does not co-operate and withdraws from the process.

### Methodology

#### Ethical approval

All participants gave written informed consent before enrolment. The protocol was approved by the Ethics Committee of Hunan Institute of Electromechanical Technology and was implemented in accordance with the Declaration of Helsinki.

Conventional rehabilitation was performed in both groups, with the addition of Nordic hamstring exercises in the test group. Both treatment groups were operated by physiotherapists. (1) Conventional rehabilitation treatment^22^. It was divided into preoperative and postoperative treatment, and the postoperative period was divided into 3 phases: early, mid and late. Preoperatively: isometric muscle contraction training of the affected limb, ankle pump exercises, active and passive joint mobility and muscle strength training of the uninjured joint and limb. Postoperative period: early (0–2 weeks postoperative), including general rehabilitation, rehabilitation of the affected limb, isometric contraction of the thigh muscles of the lower limb and ankle pump training immediately after waking up from anaesthesia, active and passive full range of joint mobility training of the adjacent joints. On the second post-operative day, passive full range of motion training of the patellofemoral joint was started, using a continuous passive motion machine (CPM) for 1 h, twice daily. The affected limb is placed flat on the bed in a deep knee position, or the posterior side of the ankle is raised by 5–10 cm to suspend the knee joint; isometric muscle training of the affected limb is performed daily, 10 sets of 3–4 sets per day, and closed chain flexion and extension muscle strength and coordination training of the affected knee are started 2 weeks after surgery.Local ice is applied after joint mobility training for 3 weeks post-operatively, along with plantar IV pump therapy and pulsed short wave therapy if the operated limb is significantly swollen. In the mid-term (3–6 weeks post-operatively), active and passive joint mobility training of the knee. Add knee resistance muscle training to maintain the early postoperative muscle training components. In the later stage (7–24 weeks after surgery), progressive resistance muscle training of the muscles around the knee joint and terminal knee extension muscle training, as well as weight transfer, gait and proprioceptive training are performed until the patient is able to walk normally. (2) Perform Nordic hamstring training^23^. Starting in the 3rd postoperative week, the patient was instructed to kneel on both knees, keep the torso upright, place the hands on either side of the torso and tense the body, the therapist secured the patient's ankles in place and applied pressure to ensure that the patient's tibia remained in contact with the mat throughout the movement, the patient's torso slowly descended forwards whilst slowing down the descent of the body through centrifugal action of the popliteal muscle group to resist the forward descending movement until it landed flat on the mat. Following the principle of over-recovering, the amount of training is appropriate for patients to have mild fatigue on the second day, and the intensity of intensive training can be appropriately increased in the later period to ensure ligament stability, such as the Nordic hamstring movement of "weighted shear squat walking".

### Efficacy evaluation criteria

#### Muscle strength tests

The patients' hamstring muscles were tested for muscle strength before treatment, at 12 weeks and 24 weeks postoperatively, respectively.

1. The test instrument is a Biodex system 3-Dynamometer operation from Biodex Medical Systems, USA, which is routinely calibrated before testing.
2. Testing modality^24^: The patient performs a 5-min warm-up activity (riding a power bike) before the test. The test is performed with the patient seated in the test chair with a 90° seat angle, the axis of the knee joint aligned with the axis of the power arm and the resistance pad at the end of the power arm fixed at 3 cm above the upper edge of the inner ankle joint.The patient's lower limbs were weighed before the test, and the range of joint movement was set from 0° to 100°. The test speed was 60°/s and 120°/s, with one unit of each speed. Five sets of maximum contraction movements were performed for each test, with an interval of 60 s between units^25^. Before the formal testing of each test speed, 3 reps of sub-polar flexion and extension exercises are performed as a warm-up, followed by the formal testing. The healthy leg is tested first, followed by the affected leg after an interval of 2 min. The average of the 5 sets of results was used as the final result.
3. Test indicators: peak torque (PT), total work (TW), average peak torque (AVG PT).

#### Functional assessment of the knee joint

The Lysholm knee score was used to assess knee function in 60 patients after week 24.

### Statistical analysis

The measurements were statistically analysed using SPSS 22.0 using a t-test, setting P < 0.05 as statistically significant.

### Informed consent

All methods were carried out in accordance with relevant guidelines and regulations. All experimental protocols were approved by the Sanya Rehabilitation and Wellness Centre. Informed consent was obtained from all subjects for all experimental protocols.

## Results

### Difference in thigh circumference between the two groups

The difference in thigh circumference was higher in the observation group than in the control group at 1 week and 1 month after surgery (P < 0.05), see Table1for details.

**Table 1:** Comparison of the difference in circumference of the thigh between the two groups ($\bar{x}$± s, cm).

Group	Number of examples	Pre-operative	12 weeks post-operative	24 weeks after surgery
Control group	30	− 1.12 ± 0.34	− 0.51 ± 0.15*	− 0.41 ± 0.34*
Test group	30	− 1.11 ± 0.32	− 0.35 ± 0.22*	0.12 ± 0.03*
T		0.137	3.847	9.943
P		0.891	0.000	0.000

*P < 0.05 compared to this group at admission.

### Muscle strength tests

The peak moments, total work, and average peak moments of the flexor and extensor muscle groups of the affected knee after ACL rupture of the patient were significantly lower than those of the healthy side at 60°/s and 120°/s, with the extensors lower than the flexors, and this was particularly prominent at the 60°/s test. At weeks 12 and 24 post-operatively, the hamstring muscle strength of both groups improved compared to the pre-operative period; the ratio of hamstring peak moment on the affected side/healthy side was significantly higher in the test group than in the control group by comparing the hamstring peak moments at different angular velocities. See Tables 2, 3 and 4.

**Table 2:** Comparison of peak moments at different testing times between the two groups of patients ($\bar{x}$± s, N m).

	N	120°/s				60°/s			
		Extension (quadriceps muscle group)		Flexion (popliteus muscle group)		Extension (quadriceps muscle group)		Flexion (popliteus muscle group)	
		Healthy side	Sick side	Healthy side	Sick side	Healthy side	Sick side	Healthy side	Sick side
Control group									
Pre-operative	30	140.27 ± 23.25^a^	98.35 ± 29.71	72.31 ± 17.88^a^	40.52 ± 18.26	186.54 ± 32.41^a^	129.22 ± 36.74	86.21 ± 22.80^a^	65.53 ± 21.60
12 weeks post-operative		148.45 ± 33.46^a^	109.78 ± 38.48	75.02 ± 23.55	56.53 ± 24.08	187.31 ± 44.17^a^	139.32 ± 40.07	88.96 ± 25.22	68.76 ± 23.16
24 weeks after surgery		153.64 ± 23.84^a^	113.67 ± 24.37	68.21 ± 16.19	61.24 ± 22.28	189.11 ± 35.74^a^	141.29 ± 37.65	92.72 ± 18.21	70.39 ± 23.81
Test group									
Pre-operative	30	154.29 ± 14.35^a^	116.26 ± 11.48	72.45 ± 10.14^a^	42.63 ± 9.22	189.45 ± 23.30^a^	132.41 ± 14.73	89.12 ± 12.33^a^	63.12 ± 13.22
12 weeks post-operative		158.17 ± 12.31^a^	127.98 ± 10.37	73.44 ± 9.91	62.20 ± 8.87	191.07 ± 20.56^a^	153.25 ± 18.03	92.86 ± 11.94	17.36 ± 12.17
24 weeks after surgery		160.18 ± 11.09^a^	142.06 ± 10.03	75.21 ± 8.44	67.98 ± 8.50	193.15 ± 18.88^a^	165.12 ± 15.32	96.61 ± 13.82	82.21 ± 11.24

^a^P < 0.05 compared to the affected side.

**Table 3:** Comparison of total work at different testing times between the two groups ($\bar{x}$± s, J).

	N	120°/s				60°/s			
		Extension (quadriceps muscle group)		Flexion (popliteus muscle group)		Extension (quadriceps muscle group)		Flexion (popliteus muscle group)	
		Healthy side	Sick side	Healthy side	Sick side	Healthy side	Sick side	Healthy side	Sick side
Control group									
Pre-operative	30	906.79 ± 116.46^a^	655.08 ± 199.15	402.28 ± 121.33^a^	304.38 ± 84.98	1074.53 ± 187.62^a^	777.24 ± 202.15	590.41 ± 123.76^a^	412.51 ± 113.15
12 weeks post-operative		908.07 ± 118.73^a^	679.55 ± 230.28	435.72 ± 116.97	339.87 ± 88.41^b^	1097.89 ± 182.03^a^	829.04 ± 199.59	591.96 ± 136.25	474.31 ± 132.21^b^
24 weeks after surgery		913.33 ± 180.01^a^	718.38 ± 140.91^b^	440.12 ± 106.18	361.62 ± 80.26^b^	1102.83 ± 175.77^a^	891.65 ± 162.43	599.92 ± 108.66	489.94 ± 120.31^b^
Test group									
Pre-operative	30	925.48 ± 102.13^a^	665.26 ± 141.61	72.45 ± 10.14^a^	42.63 ± 9.22	1135.54 ± 147.83^a^	829.11 ± 162.47	89.12 ± 12.33^a^	63.12 ± 13.22
12 weeks post-operative		928.55 ± 102.90^a^	794.24 ± 145.77	73.44 ± 9.91	62.20 ± 8.87^b^	1175.08 ± 148.63^a^	921.25 ± 146.07	92.86 ± 11.94	17.36 ± 12.17^b^
24 weeks after surgery		943.08 ± 128.91^a^	811.02 ± 113.56^b^	75.21 ± 8.44	67.98 ± 8.50^b^	1204.93 ± 149.75^a^	1048.56 ± 137.82	96.61 ± 13.82	82.21 ± 11.24^b^

Compared with the affected side,^a^P < 0.05. Compared with the preoperative side,^b^P < 0.05.

**Table 4:** Comparison of mean peak moments at different testing times between the two groups of patients ($\bar{x}$± s, N m).

	N	120°/s				60°/s			
		Extension (quadriceps muscle group)		Flexion (popliteus muscle group)		Extension (quadriceps muscle group)		Flexion (popliteus muscle group)	
		Healthy side	Sick side	Healthy side	Sick side	Healthy side	Sick side	Healthy side	Sick side
Control group									
Pre-operative	30	140.37 ± 29.60^a^	103.97 ± 32.37	66.13 ± 16.69^a^	47.25 ± 18.62	170.03 ± 33.46^a^	108.40 ± 34.65	82.21 ± 20.38^a^	60.36 ± 16.63
12 weeks post-operative		142.16 ± 29.08^a^	106.48 ± 36.27	67.62 ± 20.51	54.37 ± 18.90	174.89 ± 38.13^a^	133.64 ± 32.60	83.16 ± 22.42	64.69 ± 23.85
24 weeks after surgery		143.96 ± 29.97^a^	107.91 ± 24.89	69.17 ± 15.36	56.46 ± 17.81	175.11 ± 30.28^a^	133.55 ± 22.93	82.95 ± 18.33	65.93 ± 15.88
Test group									
Pre-operative	30	152.56 ± 29.33^a^	101.78 ± 28.32	68.17 ± 14.25^a^	48.65 ± 14.29	177.02 ± 25.13^a^	111.14 ± 27.25	86.53 ± 17.35^a^	70.52 ± 13.45
12 weeks post-operative		139.37 ± 25.93^a^	120.23 ± 26.04	76.82 ± 15.31	65.20 ± 18.41	180.28 ± 25.73^a^	156.69 ± 22.47	89.26 ± 18.47	76.63 ± 13.81
24 weeks after surgery		146.36 ± 22.11^a^	126.37 ± 20.66	77.94 ± 14.44	67.72 ± 15.62	181.02 ± 18.87^a^	159.77 ± 20.56	89.77 ± 14.91	76.82 ± 17.32

^a^P < 0.05 compared to the affected side.

### Knee examination

The test group and control group had negative Lachmen's test and anterior drawer test on recheck at week 24 postoperatively, and the difference in anterior tibial displacement was < 5 mm on the KT-1000 test and < 3 mm compared to the healthy side, with no significant difference between the two groups. See Table5for details.

**Table 5:** Lysholm knee scores at 12 and 24 weeks postoperatively ($\bar{x}$± s).

	n	Post-operative week 12	Post-operative week 24
Control group	30	66.7 ± 6.4	88.9 ± 7.9
Test group	30	82.8 ± 5.6	97.4 ± 3.0
*P*-value		0.012	0.041

## Discussion

For patients after ACL reconstruction restoration to pre-injury levels of movement and function is the ultimate goal^24^. The stability of the knee joint includes both static and dynamic stability. Static stability of the knee joint can be restored by knee arthroscopic reconstruction of the ACL with autologous allograft bone-patellar tendon-bone (BPTB)^26^. Insufficient strength of the periarticular muscles can cause dynamic instability of the knee joint, so restoring strength to the periarticular muscles is essential to stabilise the knee joint and restore function^27^. The reconstructed ligament undergoes a process of ligmentization (inflammatory response, hematologic reconstruction, fibrosis and remodelling), during which the reconstructed ligament undergoes changes from strength to weakness and then gradual strengthening^28^. The ligaments reconstructed in the early postoperative period go through a process of strength to weakness, so in order to protect the ACL from anterior displacement of the tibia the main focus is on training the hamstring muscles^29^. In the mid to late stages of surgery, Nordic hamstring exercises can facilitate ligament remodelling as the graft ligaments gradually increase in strength.

In this study, isometric muscle strength tests were performed at 12 and 24 weeks after ACL reconstruction in both groups: the muscle strength of the quadriceps and hamstrings increased in both groups compared to the preoperative period, with the hamstrings recovering to 75% of the healthy side in the control group; the hamstrings recovered to 85% of the healthy side in the test group, which was higher than the control group.This suggests that Nordic hamstring training has a significant effect on the recovery of hamstring strength after ACL reconstruction. Nordic hamstring training is a more effective, safe and accurate form of centrifugal training than other modes of plyometric training. It moves in the opposite direction to the direction of muscle contraction, and this insurmountable resistance causes the cerebral cortex and spinal cord to send more efferent impulses to the muscle to generate more force than a centripetal contraction^30^. At the same time this centrifugal stimulation helps to control and ensure the elastic component of the healing tissue at will, increasing collagen readaptation control and allowing the patient to recover maximum function^31^.

The results in Table1show that when comparing the difference in thigh circumference between the two groups of patients preoperatively and 12 weeks postoperatively, the difference in the control group was lower than that in the experimental group, suggesting that Nordic popliteal exercises were able to reduce the difference in thigh circumference in the patients.Using the circumference to evaluate the swelling, considering that in the preoperative period the influence on the circumference of the lower limb is mainly soft tissue oedema, and in the postoperative period of 12 weeks, it may be accompanied by the change of muscle atrophy, and the influencing factor on the circumference may be mainly a muscle problem^32^, so the observation period was set as 12 weeks and 24 weeks.The reason for this is that in the training mode of Nordic hamstring exercise, patients choose flexible resistance according to their own situation, which can activate the knee blood vessels and promote the flow of blood vessels in the joints of patients, thus reducing the swelling of the knee joints and reducing the difference in the circumference of the thighs of the patients, so as to keep them in line with the healthy side of the leg^33^. The results of this study showed that after treatment, the flexor-extensor PT, TW value, and AVG PT of patients in the observation group were higher than those of patients in the control group, suggesting that patients with Nordic hamstring exercises can achieve a loading pattern that matches their actual situation, which can maximise the restoration of their own leg muscle tone, and thus restore their muscle strength^34^.

From Tables2,3,4and5, it can be seen that the patient's affected knee extensor compared to flexor peak moment, total work, and average peak moment of the three test indexes were significantly reduced at 60°/s and 120°/s compared to the healthy side, in which the extensor compared to the flexor was reduced more significantly, which was particularly prominent in the 60°/s test.This may be related to the wasting muscular atrophy following ACL injury, the disruption of proprioceptive information from the ACL, the stimulation of persistent knee pain and oozing, and the disruption of ligament reconstructive surgery, which passes through the periprosthetic receptors of the knee joint to the central nervous system, potentially resulting in alterations of neural control of motor neurons and higher centres, and having a pronounced impact on periprosthetic knee muscular strength, which ultimately leads to alterations in the balance of muscular strength in the knee joint^35^. The Nordic hamstring exercise we used is a centrifugal exercise method that both improves hamstring muscle strength and reinforces signalling in the proprioceptive pathway with maximal stimulus transmission to all levels of the proprioceptive centre level^36,37^. The results of this study showed that Nordic hamstring exercises not only strengthened proprioceptive biofeedback and improved hamstring contraction, which in turn led to a balanced knee flexion and extension muscle strength.

The results in Table5show that after 24 weeks of treatment, the Lysholm knee score of the test group was significantly better than that of the control group (P < 0.05). It was shown that Nordic hamstring exercises were more effective in improving knee pain and stability after ACL reconstruction, and promoting the recovery of knee function. We found that the patients' postoperative knee stability was also significantly improved by examining all patients for clinical symptoms and signs.Even if good stability and full range of motion are restored to the knee after ACL reconstruction, the purpose of ACL reconstruction cannot be considered achieved if muscle strength is not fully restored^38^. A prospective randomised study^39^of bone-patellar tendon-bone reconstruction of the ACL, with a controlled comparison of immediate postoperative weight bearing and delayed 2-week weight bearing, found that the joint laxity was the same in both groups, and immediate weight bearing reduced the incidence of patellofemoral pain, suggesting that immediate postoperative weight bearing does not overload the ACL, and not only does it not affect the healing and stability of the graft, but it also reduces the incidence of patellofemoral pain. Beard et al.^40^concluded that the improvement of hamstring contraction capacity has a linear relationship with the recovery of knee joint function. Whereas, the enhancement of hamstring contraction capacity by Nordic hamstring exercises in this trial also ensured the kinetic stabilisation of the knee joint, which is consistent with Beard's findings.

The results of this paper show that the centrifugal moment of the quadriceps muscle also demonstrated a significant increase after 24 weeks of Nordic hamstring exercise. It has been found that the active and antagonist muscles have an interactive inhibitory effect during muscle contraction, and when the contraction of the active muscle is rapid, the antagonist muscle also experiences a synchronised contraction, and the antagonist's activity increases significantly^14^. The greater the force on the hamstrings, the greater the activation of the muscle, the improvement of the quadriceps as an antagonist muscle and the adaptation to training^41^. Therefore, the changes in centrifugal muscle strength of the quadriceps may be more related to the increased centrifugal muscle strength of the hamstrings as a result of Nordic hamstring exercises.

The limitations of this study are as follows: (1) Only 60 subjects were included. Despite the differences between individuals, the subjects were carefully selected from 92 patients with similar age and lesions. (2) The subjects' pre-disease lesion exercise habits and exercise levels were not compared, and their influence on the experimental results was not taken into account. (3) Only isometric muscle strength tests were performed on the knee joint before, 12 and 24 weeks after the intervention, and a 4th test was not performed at a later stage to determine the long-term effects of Nordic hamstring exercise on centrifugal muscle strength and stability of the knee joint.

## Conclusions

Through our clinical observation, the efficacy after ACL reconstruction is comprehensively assessed, the muscle strength deficit around the knee joint after ACL reconstruction is clarified, and a postoperative rehabilitation plan is formulated. Nordic hamstring exercises should be performed at the same time to restore muscle strength in patients with ACL ligament reconstruction, which is of great significance in restoring their normal athletic ability and preventing re-injury (Supplementary Information).

### Supplementary Information

Supplementary Information.

## Data Availability

Due to the nature of this study, participants in this study did not agree to share their data publicly and therefore supporting data could not be provided. If anyone would like to obtain data from this study, please contact the corresponding author.
