# Supplementary material for: Nordic hamstring exercises in functional knee rehabilitation after anterior cruciate ligament reconstruction: a prospective, randomised, controlled study
Source: Sci Rep. 2023 Nov 3;13:19039. doi: 10.1038/s41598-023-45817-6 (PMC10624851; doi:10.1038/s41598-023-45817-6)
Supplement: Supplementary file 1 — Supplementary Information. [file 41598_2023_45817_MOESM1_ESM.docx]

| Section/Topic | Item No | Checklist item |
| --- | --- | --- |
| Title and abstract | 1a | Nordic hamstring exercises in functional knee rehabilitation after anterior cruciate ligament reconstruction: a prospective, randomised, controlled study |
|  | 1b | OBJECTIVE: To study the effect of using Nordic hamstring exercise method on muscle strength and knee joint stability of patients after ACL reconstruction. Methods: 60 patients admitted to our hospital for ACL reconstruction were randomly divided into a test group (n=30, applying Nordic hamstring exercise) and a control group (n=30, applying conventional rehabilitation training methods), and the difference in the circumference of the thighs of the patients in the two groups was analysed after training, and the peak torque (PT) and flexion strength were measured by using the Biodex system3 Multi-Joint Isokinetic Testing System at an angular velocity of 60°/s and 120°/s of the knee joint. The peak torque (PT), total work (TW), and average peak torque (AVG PT) of the knee joint at 60°/s and 120°/s angular velocity were measured by extension and flexion strength. RESULTS: There was no difference in the difference in thigh circumference between the two groups before surgery (P＞0.05); the difference in thigh circumference in the experimental group was (-0.35±0.22) cm and (0.12±0.03) cm at the 12th and 24th weeks after surgery, respectively, which were higher than that of the control group (-0.51±0.15) cm and (-0.41±0.34) cm (P＜0.05). At the 12th and 24th postoperative weeks, the popliteal muscle strength of both groups was improved compared with that before surgery; among them, by comparing the popliteal peak moments with different angular velocities, the ratio of popliteal peak moment on the affected side/peak moment on the healthy side of the popliteal muscle of the experimental group was significantly higher than that of the control group, and the difference was significant (P＜0.05), but there was still a gap compared with that of the healthy side. The Lachmen test and the anterior drawer test were negative in the test and control groups at the 24th postoperative week of review, and the anterior tibial shift was <5 mm in the KT-1000 test, and the difference in the anterior shift was <3 mm compared with the healthy side, and there was no significant difference between the two groups. CONCLUSION: By Nordic hamstring exercise can make patients after knee ACL reconstruction reduce patient pain, accelerate the recovery of knee function, improve the swelling of the lower limb, reach the level of flexor strength of the healthy side within 24 weeks, and can increase the stability of the knee joint. |
| Introduction | | |
| Background and objectives | 2a | Injury to the anterior cruciate ligament (ACL) of the knee is one of the more serious injuries in knee sports trauma and has become a rehabilitation challenge in sports medicine, orthopaedic surgery, and rehabilitation medicine.The ACL is the predominantly restricting ligament preventing the tibia from sliding forwards relative to the femur and from internally rotating.Rupture of the ACL permits the tibia to be displaced forwards and internally rotated, which can cause recurrent episodes of knee instability and can damage the meniscus and the knee joint surface. It can cause recurrent episodes of knee instability and can damage the meniscus and knee joint surfaces. Arthroscopic reconstruction of the ACL and early postoperative rehabilitation are now commonly used to consolidate the surgical results and improve knee function after ACL rupture. In the postoperative period, patients often suffer from knee pain, combined dysfunction, oedema, muscle atrophy and other complications, and therefore require a good postoperative training system to help them recover. Studies have shown that the effect of postoperative rehabilitation greatly affects the final outcome of surgery, and the rehabilitation of muscle strength is an important part of postoperative rehabilitation for ACL reconstruction. At present, many scholars at home and abroad, using different postoperative rehabilitation training methods. Baroni et al. conducted a literature review on the methods used for centrifugal training of knee extensors in healthy subjects and assessed the adaptations of the muscle groups responsible for knee extension in terms of strength, activation and structure. Seventy-five studies were carefully analysed and 30 of them were reviewed. The results show that the increase in strength after centrifugal training of knee extensors is caused by structural adaptations in the nervous system. It may help athletes to prevent muscle injury and rehabilitation after muscle injury. Zhang Wenjie et al.'s study on ACL reconstruction patients showed that isometric centrifugal training of hamstrings can improve the patients' active knee extension angle, and enhance the muscle strength of the muscle groups related to knee flexion and knee joint function. In addition to ACL, hamstrings also play an important role in preventing anterior tibial shift. Towards the end of a fast walking stride, the hamstrings produce an effective centrifugal contraction to decelerate the calf swing, thus preventing excessive anterior tibial momentum from adversely affecting the ACL or the graft. However, postoperative hamstring muscle strength training has not been given enough attention, and most of the training methods focus on centripetal contraction, which may cause an imbalance in knee flexion and extension muscle strength and result in ACL grafts that cannot be maintained in the appropriate tension range during high-intensity exercise, leading to secondary injuries. Centrifugal training can effectively improve joint mobility, muscle strength and neuromuscular control, thus reducing the risk of injury and improving joint function. |
|  | 2b | Nordic hamstring exercise is a knee-led, self-weighted, centrifugal exercise developed by Mjolsnes et al. in 2004 and included in the "FIFA 11+" comprehensive warm-up exercises by the FIFA Medical Research Centre (F-MARC). The FIFA Medical Research Centre (F-MARC) has included the "FIFA11+" comprehensive warm-up exercises. Studies have shown that 6-10 weeks of Nordic Hamstring Exercise is effective in increasing the centrifugal strength of the hamstrings and significantly reduces the rate of injury and recurrence. Nordic hamstring exercises focus on centrifugal training for the purpose of activating the muscle, reducing the risk of endurance injuries regarding load-bearing tissues, and providing reinforcement of knee stability, preventing sports injuries, and improving lower limb mobility. In view of this, this study focuses on the effects of Nordic hamstring exercises on patients after ACL reconstruction of the knee, with the aim of finding a more effective way for patients after ACL reconstruction of the knee. |
| Methods | | |
| Participants | 3a | Sixty patients were selected after arthroscopic autologous popliteal tendon reconstruction for ACL injury from August 2018 to January 2020.To avoid other factors interfering with the test results, all the selected subjects were patients with unilateral anterior cruciate ligament rupture of the knee.All the 60 patients were males, and they were randomly divided into 2 groups of 30 cases each.①Test group ①Test group, age 29.3±6.3 years, disease duration 38.6±13.5d, 12 cases of left knee, 18 cases of right knee. ②Control group, age 31.7±4.8 years, disease duration 34.1±11.7d, left knee 9 cases, right knee 21 cases.  Inclusion and exclusion criteria  Inclusion Criteria: ① Meet the diagnostic criteria for ACL injury in the American Academy of Orthopaedic Surgeons' "Guidelines for the Treatment of Anterior Cruciate Ligament Injuries"; ② All are male, aged 18-58 years, weighing 151-187cm, 55-92kg; ③ Unilateral ACL rupture was detected by using magnetic resonance imaging and other imaging means and reconstruction was carried out; ④ Lachman's test (Lachman) or positive axial shift test; ⑤ no lower limb fracture; ⑥ no peripheral nerve injury; ⑦ no postoperative infection; ⑧ no cognitive dysfunction; ⑨ no respiratory and circulatory disorders; ⑩ signed informed consent.  Exclusion criteria: ① combined with posterior cruciate ligament rupture; ② combined with tibial and fibular collateral ligament rupture; ③ combined with meniscus tear; ④ bone - patellar tendon - bone reconstruction ACL and semitendinosus tendon reconstruction ACL patients; ⑤ one month after the operation, the range of knee flexion and extension is still unable to reach 20 ° ~ 80 ° or there are suspected to be a positive anterior drawer test; ⑥ unable to tolerate the popliteus muscle isokinetic centrifugal training; ⑦ obesity patients have difficulty in reaching 120 ° of knee angle. The angle of knee flexion is difficult to reach 120°; ⑧ history of previous knee ligament injury, femoral footprint <14mm; ⑨ bone injury, intercondylar fossa is too small and joint degeneration and other comorbidities; ⑩ osteoarthritis of the knee joint X-ray grading to reach the Ⅱ, Ⅲ, Ⅳ level lesions; ⑪ uncooperative and quit halfway. |
|  | 3b | To evaluate the difference in thigh circumference between the two groups of patients after training, the peak torque (PT), total work (TW), and average peak torque (AVG PT) were collected by using the Biodex system3 multi-joint isokinetic testing system for extension and flexion strength measurements of the knee joints at angular velocities of 60°/s and 120°/s. |
| Interventions | 4 | Conventional rehabilitation was performed in both groups, and Nordic hamstring exercises were added in the experimental group.Both treatment groups were operated by physiotherapists. ① Conventional rehabilitation treatment. Divided into pre-treatment and postoperative treatment, and postoperative is divided into early, middle and post 3 stages. Preoperative: isometric muscle contraction training of the affected limb, ankle pump exercises, active and passive joint mobility and muscle strength training of the uninjured joints and limbs. Postoperative: early stage (0~2 weeks after surgery), including general rehabilitation, rehabilitation of the affected limb, isometric contraction of the lower limb thigh muscles and ankle pump training immediately after awakening from anaesthesia, and active and passive full-range joint mobility training of the adjacent joints. Passive full range of motion training of the patellofemoral joint was started on the 2nd postoperative day, with the application of continuous passive motion (CPM) training for 1h, twice a day; the range of motion of knee flexion was 110°~130° as far as possible under the condition that the patient could tolerate it. The affected limb was placed flat on the bed in the deep knee position, or the back side of the ankle was elevated by 5~10cm to make the knee joint suspended; isometric muscle strength training was performed daily, with 10 pieces in each group, and 3~4 groups per day, and closed-chain flexion/extension muscle strength and coordination training was performed in the affected knee at 2 weeks after the operation. After 3 weeks of joint mobility training, local ice packs were applied, and plantar vein pumps were used for treatment, and pulse short-wave therapy was supplemented if the surgical limb was obviously swollen. In the middle period (3-6 weeks after surgery), the knee joint active and passive joint mobility training. On the basis of maintaining the early postoperative muscle strength training, the knee joint resistance muscle strength training was added. In the later stage (7~24 weeks after surgery), progressive resistance training of the muscles around the knee joint, terminal knee extension muscle training, etc., as well as transfer of the centre of gravity, gait and proprioception training, until the patient is able to walk normally. ② Perform Nordic hamstring exercises. Starting in the third week after surgery, the patient was instructed to kneel on both knees, keep the torso upright, hands on both sides of the torso, the body tense, the therapist fixed the patient's ankles in the appropriate position and applied pressure to ensure that the patient's tibia remained in contact with the mat throughout the exercise, the patient's torso slowly fell forward while slowing down the fall of the body through the popliteal muscle group centrifugal action to resist the forward descent of the action until the flat fall on the mat. Following the principle of recovery from overload, the training volume is appropriate for the patient to feel mild fatigue on the second day, and the intensity of the training can be increased later to ensure ligament stabilisation by increasing the intensity of Nordic hamstring movements such as "weighted shear squat walking". |
| Outcomes | 5a | 1. Muscle strength test  The muscle strength test was performed on the popliteus muscle of the patients at 12 and 24 weeks after the operation, respectively.  ①The test instrument was Biodex system3-Dynamometer operation produced by Biodex Medical Systems, USA, and the system was calibrated before the test.  ②Testing method: The patient performs a 5-minute warm-up activity (riding a power bicycle) before the test. During the test, the patient was seated in a test chair with a seat angle of 90°, the axis of the knee joint was aligned with the axis of the power arm, and the resistance pad at the end of the power arm was fixed at the upper edge of the inner ankle 3 cm from the ankle joint. Before the test, the patient's lower limbs were weighed, the range of joint movement was set from 0° to 100°, and the test speeds were 60°/s and 120°/s, with each speed being one unit. Five sets of maximal contraction exercise were performed for each test, and the interval between two units was 60 s. Before the formal test of each test speed, 3 times of subpolar flexion and extension exercises were performed as warm-up activities, and then the formal test was performed. The healthy leg was tested first, followed by the affected leg after a 2-minute interval. The average of the 5 sets of results was used as the final result.  (iii) Test indexes: peak torque (PT), peak torque to body weight ratio (PT/BW), total work (TW), average peak torque (AVG PT).  2. Knee function assessment  Knee function was assessed in 60 patients after week 24 using the Lysholm knee score. |
|  | 5b | 1. Difference in thigh circumference between the two groups of patients  The difference in thigh circumference in the observation group was higher than that in the control group at 12 weeks and 24 weeks postoperatively (P < 0.05).  2. Muscle strength test  The peak moments, total work and average peak moments of the flexor and extensor muscles of the affected knee were significantly lower than those of the healthy side at 60°/s and 120°/s after ACL rupture, with the extensor muscles lower than the flexor muscles, especially at 60°/s. The peak moments, total work and average peak moments of the flexor and extensor muscles of the affected knee were lower than those of the healthy side at 60°/s. At the 12th and 24th postoperative weeks, the popliteus muscle strength of both groups was improved compared with that of the preoperative period; among them, by comparing the popliteus peak moments at different angular velocities, the ratio of the popliteus peak moment on the affected side of the test group to the peak moment on the healthy side of the test group was significantly higher than that of the control group, and the difference was significant (P<0.05), but there was still a gap between the test group and the healthy side.  3. Knee joint examination  The Lachmen test and the anterior drawer test were negative in the test group and the control group at the 24th week after the operation, and the anterior tibial shift was <5mm in the KT-1000 test, and the difference in the anterior shift was <3mm compared with that of the healthy side, and there was no significant difference between the two groups. |
| Randomisation | | |
| Sequence genertation | 6a | Randomised into two groups using random number table method |
|  | 6b | Age and disease duration were used as stratification factors for the trial, and postoperative ACL patients enrolled in the trial were first stratified and then randomised into groups. |
| Implementation | 7 | Generation of random allocation sequence, enrolled subjects and those who randomly allocated subjects to the intervention |
| Statistical methods | 8 | Measurements were statistically analysed using SPSS 22.0 using the t-test, setting P < 0.05 as statistically significant. |
| Results | | |
| Recruitment | 9 | Recruitment Objects  Meet all the following conditions:  1. Age 18~58 years old.  2. Unilateral anterior cruciate ligament injury without other ligament tears  3. willing to cooperate with postoperative rehabilitation and follow-up, voluntarily participate in the trial and sign the informed consent form.  And do not meet any of the following exclusion conditions:  1. Anterior cruciate ligament revision surgery.  2. patients with other ligament injuries (posterior cruciate ligament, medial or lateral collateral ligament injuries).  3. patients with neuromuscular insufficiency (e.g., paralysis, rhabdomyolysis, or muscle weakness) that would result in postoperative knee instability or gait abnormalities.  4. Patients with a previous history of knee surgery.  5. Active infected lesions in the knee joint or other parts of the body.  6. Severe osteoporosis, metabolic bone disease, radiological bone disease, tumour around the knee joint. |
| Numbers analysed | 10 | The number of subjects included was based on the original allocation group for the |
| Discussion | | |
| Limitations | 11 | The limitations of this study are as follows: 1) Only 60 subjects were included. Despite the differences between individuals, the subjects were carefully selected from 92 patients with similar age and lesions. (ii) The subjects' pre-disease lesion exercise habits and exercise levels were not compared, and their influence on the experimental results was not taken into account. (iii) Only isometric muscle strength tests were performed on the knee joint before, 12 and 24 weeks after the intervention, and a 4th test was not performed at a later stage to determine the long-term effects of Nordic hamstring exercise on centrifugal muscle strength and stability of the knee joint. |
| Generalisability | 12 | A comprehensive assessment of the efficacy of ACL reconstruction, clarification of the muscle strength deficits around the knee joint after ACL reconstruction, and development of a postoperative rehabilitation programme should be accompanied by Nordic hamstring exercises to restore muscle strength in patients undergoing ACL ligament reconstruction, which is important for the restoration of normal athletic performance and prevention of re-injury. |

Consort Flow Diagram

Assessed for eligibility (n=128)

Enrollment

Excluded (n=68)

- Not meeting inclusion critera (n=36)
- Declined to participate (n=0)
- Other reasons (n=32)

Randomized （n=60）

Allocation

Allocated to Control group (n=30)

- Received allocated Control group (n=30)
- Did not receive allocated intervention (n=0)
- Conventional rehabilitation treatment

Allocated to Test group (n=30)

- Received allocated Test group (n=30)
- Did not receive allocated intervention (n=0)
- Conventional rehabilitation treatment + Perform Nordic hamstring training

Allocation

Observation index

 Difference in thigh circumference of patients in the group after training

 The peak torque (PT), peak torque to body weight ratio (PT/BW), total work (TW), and average peak torque (AVG PT) were measured by using Biodex system3 multi-joint isokinetic test system for extension and flexion strength of the knee joints at angular velocities of 60°/s and 120°/s.

 Knee function was assessed in 60 patients using the Lysholm knee score.

Statistical analysis

Measurements were statistically analysed using SPSS 22.0 using the t-test, setting P < 0.05 as statistically significant。

in the end

Conclusion.

Through our clinical observation, we have made a comprehensive assessment of the efficacy of ACL reconstruction, clarified the situation of muscle strength defects around the knee joint after ACL reconstruction, and formulated a postoperative rehabilitation plan, which should be accompanied by Nordic popliteal muscle exercises to restore the muscle strength of patients with ACL ligament reconstruction, which is of great significance for the restoration of normal athletic ability and the prevention of re-injury.
